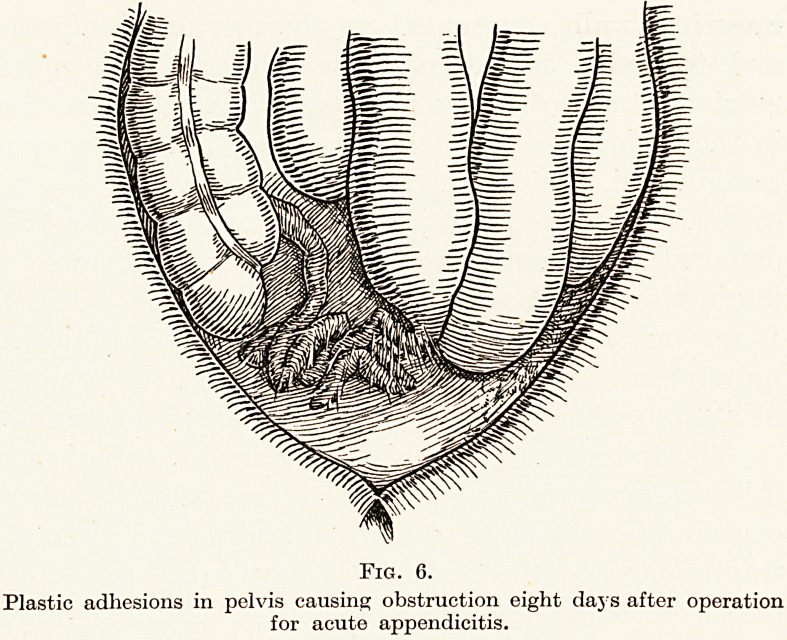# Acute Intestinal Obstruction
*An address given to the Bristol Medico-Chirurgical Society, February 12th, 1930.


**Published:** 1930

**Authors:** D. P. D. Wilkie

**Affiliations:** Professor of Surgery, University of Edinburgh


### The Bristol Medico-Chirurgical Journal

*" Scire est nescire, nisi id me*
*Scire alius sciret."*

SUMMER, 1930.

### ACUTE INTESTINAL OBSTRUCTION.*

BY

D. P. D. Wilkie, O.B.E., M.D., Ch.M., F.R.C.S., F.R.S.E.,

*Professor of Surgery, University of Edinburgh.*

There are no cases in medical practice so dramatic and so urgent in their appeal for relief as those of intestinal obstruction. Opinions may differ as to the correct line of treatment to be followed in many diseases, but for a mechanical obstruction of the bowel there can be only one treatment, namely surgical intervention. Timely operation should save most sufferers from intestinal obstruction, yet the death-rate from this condition is still well-nigh 40 per cent., and many of the victims represent robust and valuable lives. In many of the fatal cases no blame can attach to the practitioner or surgeon who dealt with them, but solely to the fact that medical help was sought too late. In a few a wrong diagnosis may have led to fatal delay, as when a strangulated femoral hernia has been missed or an internal strangulation has been treated as simple colic. In a number an apparently successful operation

* An address given to the Bristol Medico-Chirurgical Society, February 12th, 1930.

H

has been done for the relief of the obstruction, but death, possibly from toxæmia, has occurred some days after operation. Whilst recognizing, therefore, that early diagnosis and prompt surgical treatment will always form the best safeguards for the patient, we may with advantage study the factors which make for a fatal issue in such cases, that our treatment may be directed to combat these and be based on sound scientific data.

A great amount of experimental work has been done in regard to the cause of death in intestinal obstruction. The subject is a very difficult and complicated one, because of the many factors which come into play in varying degrees in individual cases. The functions of different parts of the intestinal tract are so diverse, the inter-relation of function of one part to another so imperfectly understood, the presence or absence of tension and interference with blood supply so important, that before inferences can be drawn from any experimental findings a very critical examination of the bearing of all such factors must be made. We now realize that peritonitis, once thought to be the ultimate cause of death in obstruction, is only of importance in cases where devitalization of a portion of gut from vascular occlusion or from tension of confined content is operative. The toxic character of the intestinal content above an obstruction has been determined most often by the intravenous injection of filtrates, a standard by which normal intestinal content is hardly less toxic.

Whilst emphasizing the need for care in interpreting experimental results, we must acknowledge the debt which surgery owes to the research worker in this field. Experiment has borne out completely our clinical experience that high obstruction is much more rapidly fatal than is low, and has allowed of a controlled study of the blood chemistry. The work of recent years has tended ever more fully to establish that a profound interference with intestinal physiology is the most important factor in simple obstruction and overshadows the pathological changes, whilst in strangulation and in fæcal—containing closed-loop obstruction the pathological changes in the gut determine the fatal issue.

Among the great variety of pathological conditions responsible for intestinal obstruction we may distinguish certain fundamental types. These are :—

1. Simple obstruction of the intestinal lumen.
2. Closed loop obstruction.
3. Interference with the vascular supply of the intestine.
4. Strangulation.

A fifth type, the so-called adynamic ileus of peritonitis, will also be discussed. In a number of cases these types may be combined, nevertheless they must be analysed separately.

*Simple Obstruction.*—By this we mean that the continuity of the lumen of the bowel is obstructed, either by a foreign body, *e.g.* a gall-stone, by the final occlusion of an organic stricture—tuberculous or malignant—by a kink or pressure from a band or adhesion, or occasionally by pressure from a tumour from without. The urgency of the condition varies inversely with the distance from the upper end of the intestinal tract. Thus occlusion of the distal end of the duodenum may prove fatal in sixty hours, whilst occlusion of the distal colon may be survived for two weeks. The essential difference between the high and the low obstruction would appear to be the loss of all the digestive secretions by vomiting which attends the former, and which is but a late event in the latter.

Whenever we plant an obstruction between the upper secretory part of the intestinal canal and the lower absorptive reaches, we deal a shrewd blow to the whole physiological mechanism of the digestive tract, with immediate and dramatic results. (Fig. 1.) Whilst

FIG. 1.
Arrows represent secretion into and absorption from gut.
Obstruction at A entails loss of secretion.

it is possibly true that toxic materials of the nature of proteoses may form in and may be absorbed from the bowel above the obstruction, it is equally true that the content of the obstructed bowel produces no toxic symptoms when introduced into the intestine below.

Whilst, therefore, at operations for simple obstruction it may, on mechanical grounds, seem expedient to evacuate the dilated and distended coils above the obstruction, we are losing content which might be of real value in the empty coils below. In such cases, apart from the actual mechanical relief which operation alone can give (if we except acute duodenal ileus where posture may relieve the obstruction), we must endeavour to make good the loss due to continued vomiting of secretions.

Dehydration is undoubtedly a very important factor. Hartwell showed that dogs lose as much as 5 per cent. of their body weight daily from vomiting when suffering from a high intestinal obstruction. Further, there is invariably a rise in the non-protein nitrogen of the blood in obstruction, sometimes amounting to six or seven times the normal. This rise may be accentuated during the first twenty-four hours following relief of the obstruction. Abundant fluid is an obvious need, and generous and repeated subcutaneous infusions must be given.

Some years ago Haden and Orr drew attention to the pronounced loss of blood chlorides in cases of high obstruction, the vomited gastric secretions apparently accounting for the loss. Replacement of chloride loss is thus another need, and a further reason for saline infusions. As Hartmann and Elman have pointed out, however, the loss of pancreatic and intestinal secretions introduces another element which cannot be ignored. From a study of the blood chemistry they conclude that a " combined solution " containing the chlorides of sodium, potassium and calcium, and sodium lactate, is necessary to restore normal conditions.

An important addition to our post-operative therapeutic agents is the use of hypertonic saline given intravenously. Injected very slowly, 20 c.c. of 15 per cent. sodium chloride is given into the median basilic vein. The rationale of this treatment is as follows : A rapid replacement of chloride loss is effected. Further, as Hughson and Scarff have shown, intestinal absorption is lessened, whilst at the same time vigorous peristalsis is induced. These observers demonstrated that hypertonic saline was more effective in instigating peristalsis than was pituitrin, and was thus of value, not only in promoting the downward passage of content after relief of mechanical obstruction, but also in cases of adynamic ileus and post-operative gaseous distension. An effort to replace the loss of the biliary and pancreatic secretions by giving the vomited material as an enema is rational and probably useful, and the work of Brockman on the value of enemata of bile in paralytic ileus is interesting in this connection.

The part played by bacteria in elaborating in the distended bowel above the obstruction a toxic substance which is absorbed and causes systemic poisoning is not yet fully determined. That *B. Welchii* is found in great numbers in the obstructed jejunum is undoubted, and the injection of *B. Welchii* antiserum, as recommended by Williams, to combat toxæmia may well be used as an adjuvant to the physico-chemical measures already mentioned. Clinically I have seen no impressive results from its use.

It is true that in some cases a wide exposure at operation may be necessary to find and relieve the obstruction, and a partial evisceration may even be required. On the other hand, the least exposure of viscera consistent with displaying the obstruction should be practised, and this may usually be done by finding the collapsed bowel below, and tracing it up whilst the distended coils are confined within the abdomen. In late cases of both high and low obstruction, where greatly distended and engorged jejunal coils are encountered, not only should they be emptied at operation, but a valvular tube-jejunostomy should be left, so that free drainage and lavage may be instituted and nourishment introduced.

It is almost unnecessary to state that where an organic stricture is found to be the cause of the obstruction, it should be left for late resection, and drainage of the bowel above should constitute the only immediate procedure—an ileostomy for tubercle of the small, a cæcostomy for carcinoma of the large intestine.

*Closed Loop Obstruction.*—The confinement of intestinal content within a closed loop is ever a source of grave danger. This type of obstruction forms a factor in all cases of strangulated hernia, and in internal strangulations, but is seen in its purest form when the vermiform appendix has its lumen obstructed by the impaction of a concretion in a stenosed area, or at a kink due to a peritoneal fold or an adhesion. The profession has been slow to recognize this form of obstruction, and to distinguish it from simple inflammation of the appendix, with which it is so commonly classed. Until this distinction is clearly drawn by those who teach pathology and surgery, we cannot hope to lower the death-rate from appendicitis so-called.

That the confinement of fæcal matter within a closed loop of intestine or an appendix with its proximal end occluded will lead inevitably to putrefaction, tension and gangrene is not yet generally understood and realized as a fundamental fact in pathology. The experiments with closed loops of duodenum and upper jejunum have little bearing on the type which I wish to emphasize. It is the presence of bacteria-laden organic matter undergoing putrefaction which places appendicular obstruction in a class by itself. A closed loop of small intestine as a rule contains little organic matter to decompose. Fill it, however, with cæcal content, and it will be gangrenous within twenty-four hours. (Fig. 2.) Acute obstruction of the appendix, with its typical clinical picture of colicky pain and vomiting, whilst the pulse-rate and temperature remain normal, is overlooked more frequently than any other acute abdominal disease, and is yet the commonest form of intestinal obstruction. (Fig. 3.) The difference between removing such an obstructed appendix before the tense, gangrenous wall has given way and after perforation has occurred, with the voiding of its stinking fæculent content into the peritoneal cavity, is the difference between safety and imminent danger to life. Acute inflammation of the appendix is a relatively harmless disease, acute obstruction of the appendix is one of the most fatal of diseases if not promptly dealt with.

*Interference with Blood Supply.*—Embolism or thrombosis of a mesenteric vessel gives rise to a potential obstruction in the segment of the gut involved. (Fig. 4.) The essential factors of danger in this case, however, are the lowered resistance of the intestinal wall, permitting of the passage of bacteria with consequent peritoneal infection, and the absorption of autolytic products from the damaged devitalized tissues. The presence of recognized heart disease may facilitate the diagnosis, and a rapid resection of the devitalized intestine offers the only chance of recovery.

*Strangulation.*—In this, the most dangerous of all forms of obstruction, several factors are at work, each of which in itself might prove fatal. The vascular supply of a segment of gut is interfered with, a closed loop of intestine is formed, and the intestinal passage

### PLATE I.

Fig. 2.
Closed loop of ileum filled with fæcal matter in cat. Tension gangrene of loop within 24 hours.

Fig. 3.
Acute appendicular obstruction. Gangrenous retrocæcal appendix exposed. Inset, appendix opened showing obstructing concretion.

PLATE II.

FIG. 4.
Thrombosis of branch of superior mesenteric artery causing potential obstruction.

FIG. 7.
Fatal obstruction in rabbit due to recent (48 hours) plastic adhesion. Note distended jejunum and collapsed coils of ileum.

is occluded. A fourth and most important factor is also present, namely the twist or compression of the mesentery with resultant shock which may dominate the clinical picture. (Fig. 5.) The patient indeed may be moribund whilst peritoneal infection is still negligible and absorption of toxins and autolytic products but slight. The call for immediate operation is thus more clamant in this than in any other form of obstruction, and frequently a resection will be required. The advantages of spinal anæsthesia in such cases should be remembered, and the need for fluid and chlorides is no less than in simple obstruction, as persistent and copious vomiting is a feature of all strangulations.

*Paralytic Ileus.*—It is now beyond doubt that many patients suffering from acute peritonitis die, not so much from the peritoneal infection *per se*, as from the effects of super imposed intestinal obstruction. It has been customary to designate such obstruction paralytic or adynamic, and to postulate that paresis of the intestinal wall, consequent on the peritoneal infection and toxæmia, is the underlying cause. The post-mortem study of many such cases convinced me that in the majority a mechanical obstruction was present and, indeed, that at the time of death no microscopic evidence of active infection in the peritoneum could be demonstrated, in other words, that the patients recovering from peritonitis had died from obstruction. (Fig. 6.) It was not difficult to demonstrate experimentally that quite recent fibrinous adhesions may, in the presence of a minimal infection, give rise to fatal obstruction.

The abdomen of a rabbit was opened, a coil of small intestine was gently rubbed with gauze and an emulsion containing one-fiftieth of a lethal dose of staphylococcus was applied to the coil. Two days later the animal died from acute intestinal obstruction. A recent plastic

FIG. 5.

Strangulation of bowel. A—Distended bowel above obstruction. B—Closed loop. C—Interruption of circulation. D—Compression of mesentery.

FIG. 6.

Plastic adhesions in pelvis causing obstruction eight days after operation for acute appendicitis.

adhesion had determined the obstruction. (Fig. 7.) Cultures from the peritoneum proved sterile. Repeated on several occasions, this experiment gave similar results. When, after rubbing with gauze and infecting the surface of the loop, sterile liquid paraffin was applied fatal obstruction did not occur.

Two methods of dealing with such obstructions suggested themselves. The one was to explore the abdomen, breaking down recent adhesions and endeavouring to prevent their reformation by smearing the surface with an oily substance. Sterilized liquid paraffin was the substance recommended. The other method was to perform a temporary enterostomy to relieve the distension of the obstructed gut, and give it a chance to recover tone whilst the natural resolution of the plastic adhesions occurred. Both methods proved successful in practice, but the latter was the much more generally applicable, and therefore to be preferred.

In 1914 Sampson Handley published his paper on "Ileus Duplex," in which he again emphasized the true obstructive nature of many of these cases, but added the factor of colon obstruction due to the œdema and local paresis of the pelvic portion of the colon in the inflamed zone of peritoneum. The treatment recommended by him, namely jejuno-colostomy and cæcostomy, has undoubtedly saved many lives, but is more complicated than the simple enterostomy and, in my opinion, no more successful. Much has been written in support of the value of enterostomy in post-peritonitic obstruction, and where peritonitis had been diffuse and lower abdominal in type and not generalized, it is unquestionably a valuable life-saving measure.

A group of cases is left, however, where the peritoneal infection has been generalized, where a true paresis is present, where clinically the absence of all colicky pain raises little suspicion of any organic obstruction, and where enterostomy, except as an inlet for lavage and for nourishment, gives little benefit. In such cases fomentations, subcutaneous saline, morphia, and possibly bile enemata, indeed the old treatment of peritonitis, offer as much hope as any surgical measure. Clinically the important point is to distinguish the true mechanically obstructed cases, usually associated with pelvic peritonitis, and accompanied by colicky pains, from the true adynamic type. In all of the former, and in doubtful cases, the patient should be given the benefit of a jejunostomy.

*Diagnosis.*—Whilst it is impossible to discuss this question fully, there are certain points which experience has taught me require emphasis. The all-important question, in the presence of a case of acute abdominal colic with vomiting, arises: Is there an organic obstruction of the intestine requiring immediate operation, or is the trouble one which brooks delay? The presence of shock, if renal colic be excluded, will leave little doubt that some form of strangulation has occurred and operation is urgently called for. If the patient is the subject of valvular heart disease, an embolism may be suspected. Acute pancreatitis may simulate a strangulation, but requires equally prompt surgical intervention. The type of patient, a history of biliary trouble, slight lividity of the skin, and epigastric tenderness and fulness will suggest pancreatitis. It may seem impertinent to remind you to examine carefully for hernia in all cases, and especially femoral hernia, but few of us are blameless in this matter. If a small femoral swelling be present, no matter how painless and insignificant in size, it is almost certainly the cause of the symptoms, and should be dealt with. It should be remembered that abdominal distension is usually absent during the early stages of all cases of high intestinal obstruction, and that behind a muscular abdominal wall several distended jejunal coils may be readily concealed. The presence of intestinal splashing on dipping palpation is pathognomonic of obstruction, and is an indication for surgical interference.

An obstructed appendix may give few local signs, but if intermittent colic and vomiting have occurred and slight tenderness and rigidity be found in the iliac region, no delay in operating is justifiable.

In cases where symptoms have been present for some days, and abdominal distension exists when the patient is first seen, diagnosis is often more difficult. The continuance of colic-like pains with obstipation points to an organic obstruction which may be in the colon. If colic be absent, a peritonitis of appendicular or pelvic origin is likely.

It must not be forgotten that an acute obstructive cholecystitis in old people may give rise to obstipation with abdominal distension—mainly right-sided and cæcal—and simulate closely that due to carcinoma of the colon, nor must the flatulent distension associated with a stone in the ureter be overlooked. Such cases, however, whilst they may challenge diagnosis, are seldom neglected. It is the early case, with impressive symptoms but few physical signs, which is apt to escape diagnosis.

### Surgical Treatment.

Whilst prompt interference is rightly described as the fundamental factor of success, hurry may result in disaster. When a dehydrated patient, who has been vomiting fæculent material, is brought straight to the operating theatre and anæsthetised, avoidable risks are being taken. Vomiting may result in drowning or aspiration pneumonia. A heart acting feebly on a diminished blood volume may give out ere the operation is completed. A pre-operative delay of thirty to forty minutes, during which two pints of saline are given subcutaneously and the stomach is washed out, should be a standard procedure in these cases.

*Anæsthesia.*—An operation which is so often exploratory, in so far as the true nature of the cause of obstruction is not known, may appear to demand a general anæsthetic. The advantages of spinal anæsthesia must not, however, be overlooked. The reduction of shock and the perfect relaxation obtained by this method are strong points in its favour, and if a skilled anæsthetist be not at hand, it is unquestionably the method of choice. Where the obstruction is obviously due to a tumour of the colon, a local anæsthetic will suffice for a cæcostomy.

Operative technique does not concern us here. It will suffice to say that the less that is done, consistent with relieving the obstruction and leaving no devitalized bowel behind, the better. In the after-treatment the continued need for fluid containing salt and glucose, the benefit from hypertonic salt solution given intravenously, and the value of the stomach tube, must ever be kept in mind.

In conclusion I would emphasize :—

1. The great responsibility which a medical man takes in advocating anything but immediate surgical interference in cases of doubtful intestinal obstruction.

2. The necessity for recognizing, and dealing promptly with, cases of acute appendicular obstruction.

3. The importance of making good dehydration by pre- and post-operative subcutaneous saline infusions.

4. The value of hypertonic saline intravenous injections to replace the loss of chlorides and stimulate peristalsis.

5. The value of pre- and post-operative gastric lavage.

6. The value of spinal anæsthesia, particularly in cases of strangulation associated with shock.

## Figures and Tables

**Fig. 1. f1:**
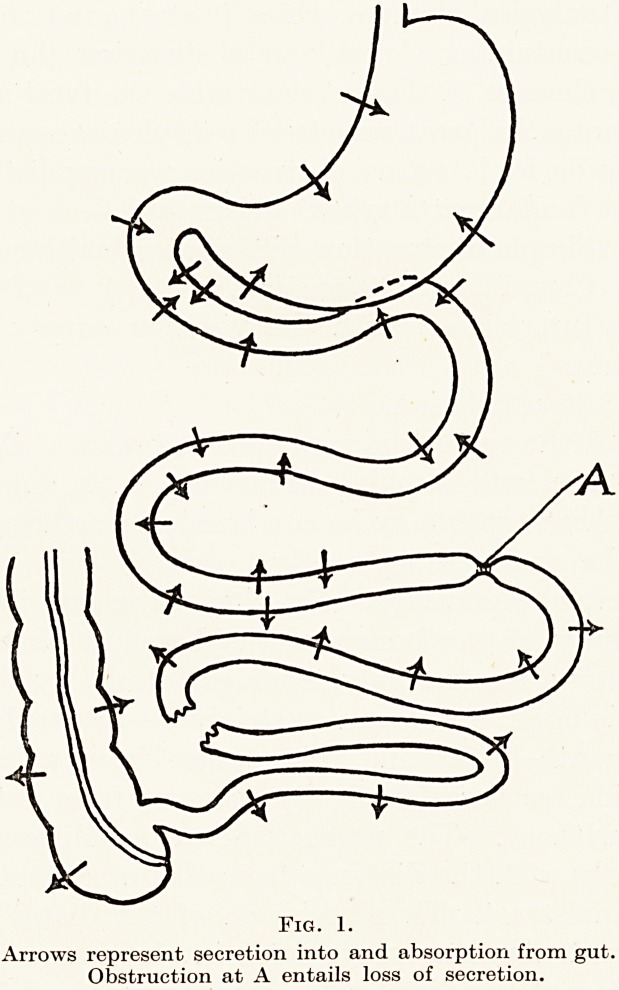


**Fig. 2. f2:**
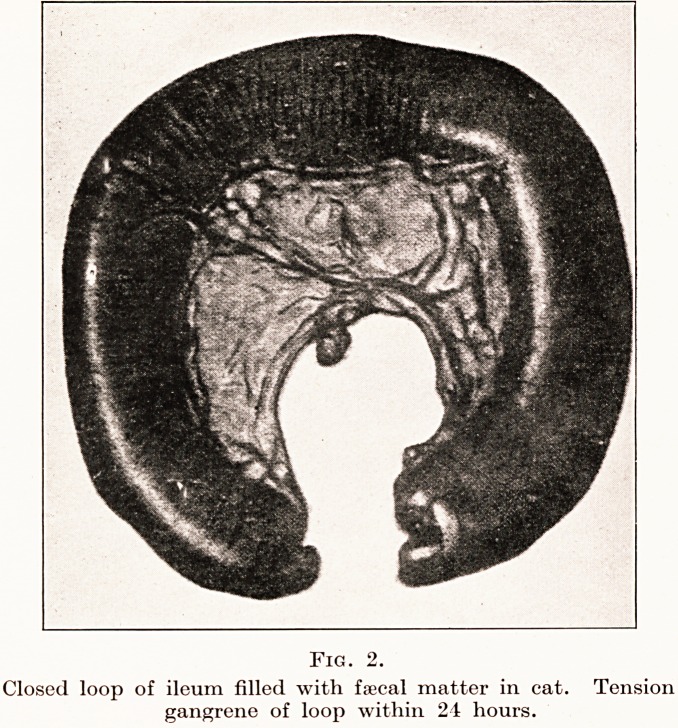


**Fig. 3. f3:**
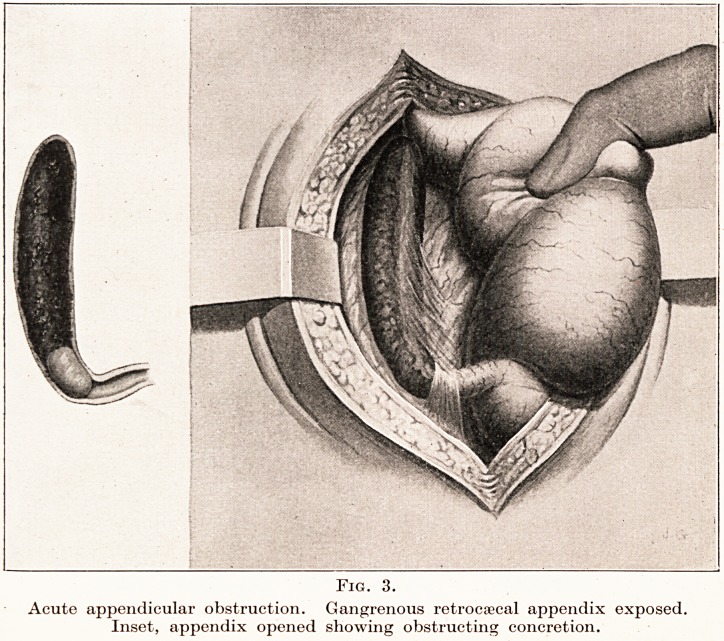


**Fig. 4. f4:**
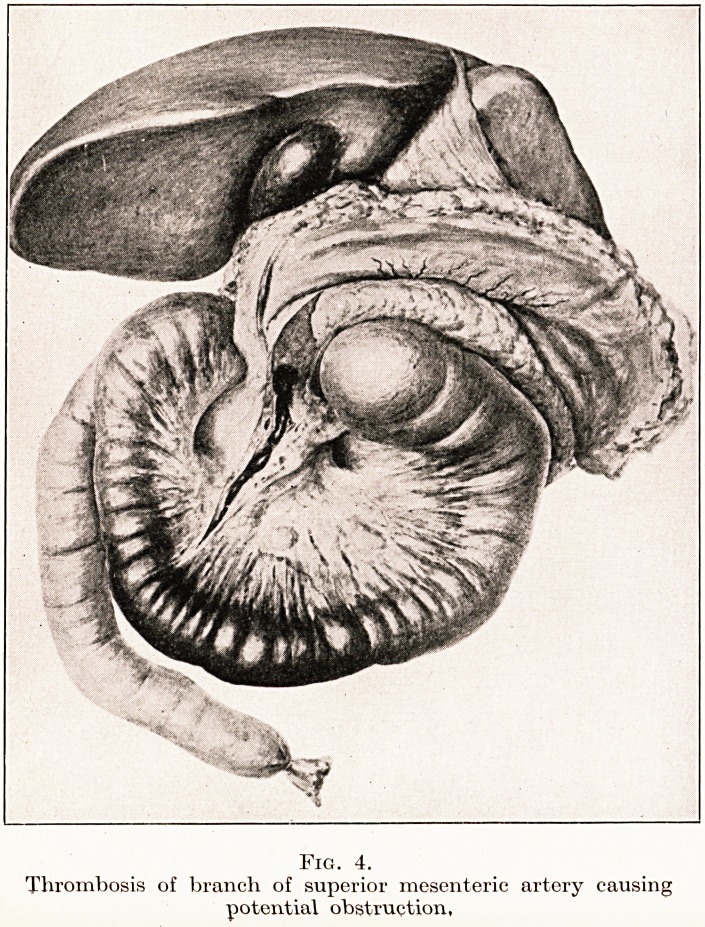


**Fig. 7. f5:**
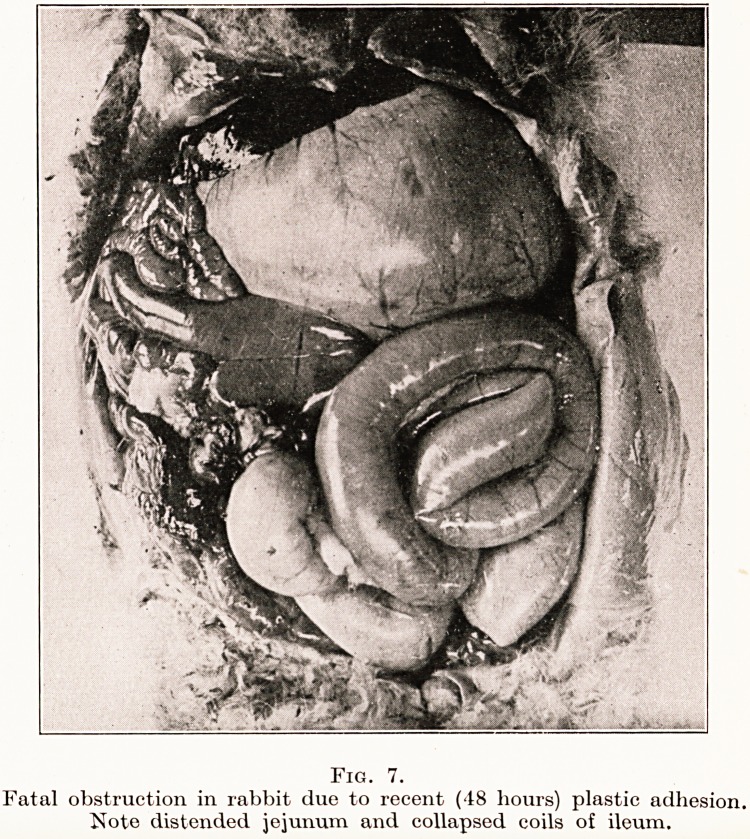


**Fig. 5. f6:**
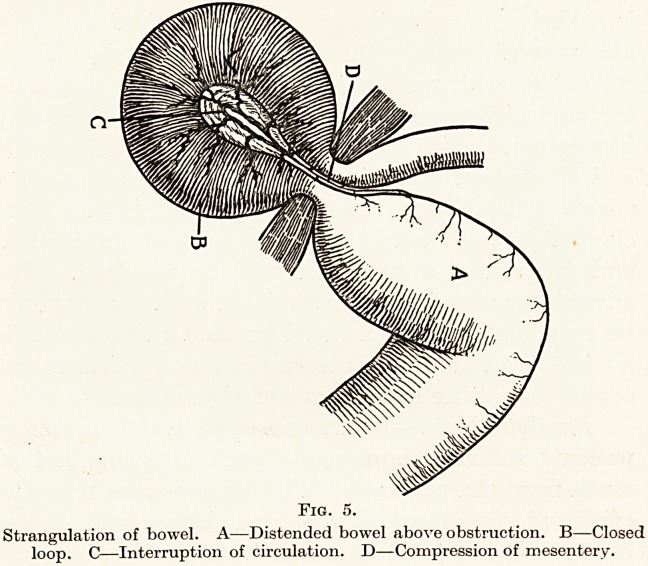


**Fig. 6. f7:**